# Developmental transcriptomes of the sea star, *Patiria miniata*, illuminate how gene expression changes with evolutionary distance

**DOI:** 10.1038/s41598-019-52577-9

**Published:** 2019-11-07

**Authors:** Tsvia Gildor, Gregory A. Cary, Maya Lalzar, Veronica F. Hinman, Smadar Ben-Tabou de-Leon

**Affiliations:** 1 Department of Marine Biology, Leon H. Charney School of Marine Sciences, University of Haifa, Haifa, 31905 Israel; 2 Departments of Biological Sciences and Computational Biology, Carnegie Mellon University, Pittsburgh, PA 15213 USA; 3 Bionformatics Core Unit, University of Haifa, Haifa, 31905 Israel

**Keywords:** Evolutionary developmental biology, Evolutionary genetics, Gene expression

## Abstract

Understanding how changes in developmental gene expression alter morphogenesis is a fundamental problem in development and evolution. A promising approach to address this problem is to compare the developmental transcriptomes between related species. The echinoderm phylum consists of several model species that have significantly contributed to the understanding of gene regulation and evolution. Particularly, the regulatory networks of the sea star, *Patiria miniata* (*P*. *miniata*), have been extensively studied, however developmental transcriptomes for this species were lacking. Here we generated developmental transcriptomes of *P*. *miniata* and compared these with those of two sea urchins species. We demonstrate that the conservation of gene expression depends on gene function, cell type and evolutionary distance. With increasing evolutionary distance the interspecies correlations in gene expression decreases. The reduction is more severe in the correlations between morphologically equivalent stages (diagonal elements) than in the correlation between morphologically distinct stages (off-diagonal elements). This could reflect a decrease in the morphological constraints compared to other constraints that shape gene expression at large evolutionary divergence. Within this trend, the interspecies correlations of developmental control genes maintain their diagonality at large evolutionary distance, and peak at the onset of gastrulation, supporting the hourglass model of phylotypic stage conservation.

## Introduction

Embryo development is controlled by regulatory programs encoded in the genome and executed during embryogenesis^1^. Genetic changes in these programs that occur in evolutionary time scales lead to alterations in body plans and ultimately, to biodiversity^1^. Comparing developmental gene expression between diverse species can illuminate evolutionary conservation and changes that underlie morphological similarity and divergence. To understand how developmental programs change with increasing evolutionary distances it is necessary to compare closely related and further diverged species within the same phylum.

**Figure 1:**
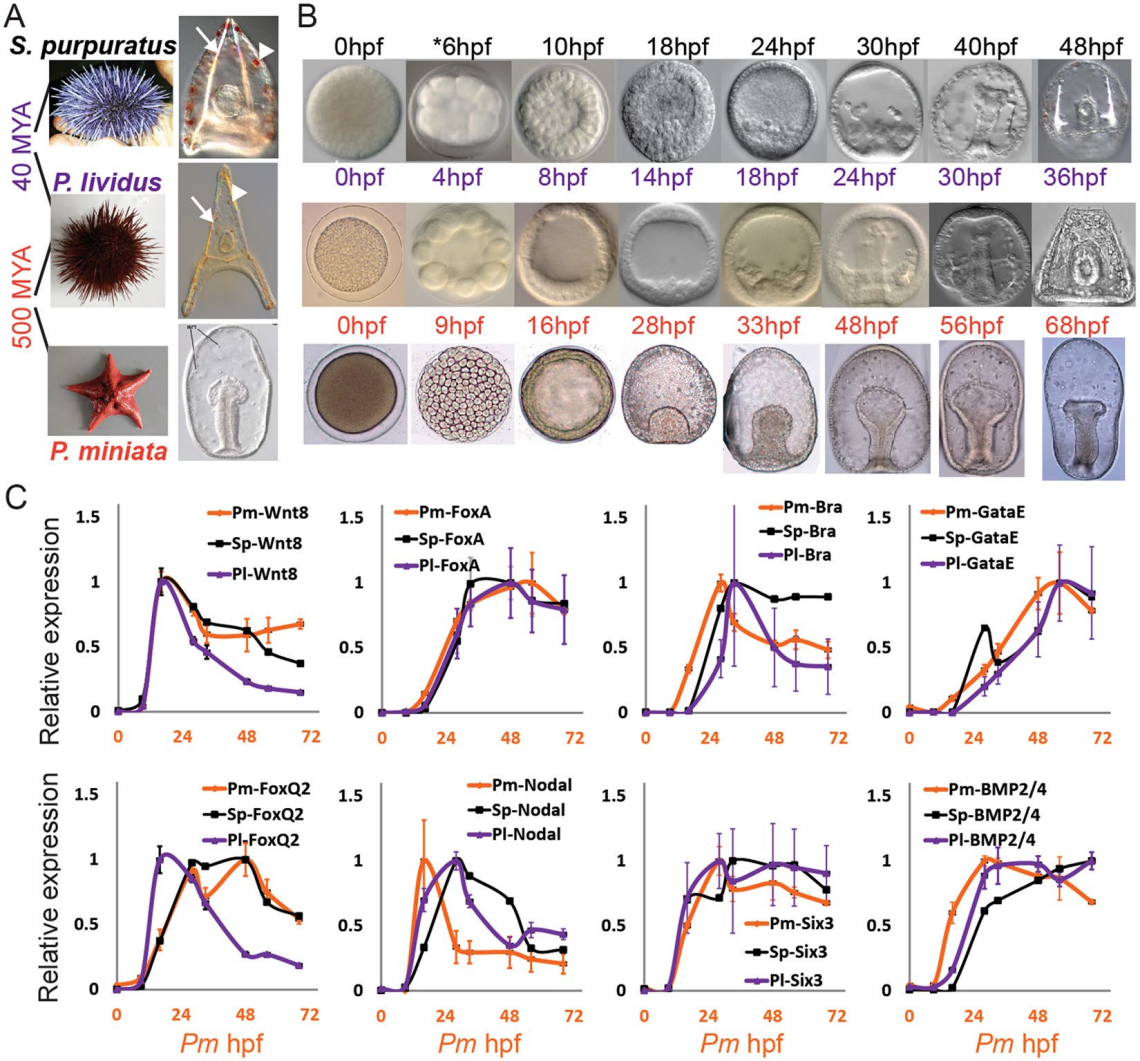
Developmental time points studied and examples for gene expression profiles on the three species. (**A**) Images of adult and larval stage of *P*. *miniata*, *P*. *lividus* and *S*. *purpuratus.* Arrows point to the sea urchin skeletogenic rods and arrow heads point to the sea urchin pigments. (**B**) Images of *P*. *miniata*, *P*. *lividus* and *S*. *purpuratus* embryos at the developmental stages that were studied in this work. Time point 6 hpf in *S*. *purpuratus* does not have RNA-seq data. (**C**) Relative gene expression in the three species measured in the current paper by RNA-seq for *P*. *miniata* (orange curves), by RNA-seq for *P*. *lividus*^19^ (purple curves) and by nanostring for *S*. *purpuratus*^28^ (black curves). Error bars indicate standard deviation. To obtain relative expression levels for each species we divide the level at each time point in the maximal mRNA level measured for this species in this time interval; so 1 is the maximal expression in this time interval.

The echinoderm phylum provides an excellent system for comparative studies of developmental gene expression dynamics. Echinoderms have two types of feeding larvae: the pluteus-like larvae of sea urchins and brittle stars, and the auricularia-like larvae of sea cucumbers and sea stars^2^. Of these, the sea urchin and the sea star had been extensively studied both for their embryogenesis and their gene regulatory networks^3–13^. Sea urchins and sea stars diverged from their common ancestor about 500 million years ago, yet their endoderm lineage and the gut morphology show high similarity between their embryos^6^. On the other hand, the mesodermal lineage diverged to generate novel cell types in the sea urchin embryo (Fig. 1A)^8,13,14^. Specifically, the skeletogenic mesoderm lineage, which generates the larval skeletal rods that underlie the sea urchin pluteus morphology^13^, and the mesodermal pigment cells that give the sea urchin larva its red pigmentation (arrow and arrowheads in Fig. 1A). Thus, there are both morphological similarities and differences between sea urchin and sea star larval body plans that make these classes very interesting for comparative genetic studies.

The models of the gene regulatory networks that control the development of the sea urchin and those that control cell fate specification in the sea star are the state of the art in the field^3–11^. The endodermal and ectodermal gene regulatory networks show high levels of conservation between the sea urchin and the sea star in agreement with the overall conserved morphology of these two germ layers^6,9,11^. Surprisingly, most of the transcription factors active in the skeletogenic and the pigment mesodermal lineages are also expressed in the sea star mesoderm^7,8^. The expression dynamics of key endodermal, mesodermal and ectodermal regulatory genes were compared between the Mediterranean sea urchin, *Paracentrotus lividus* (*P*. *lividus*) and the sea star, *Patiria miniata* (*P*. *miniata*)^15^. Despite the evolutionary divergence of these two species, an impressive level of conservation of regulatory gene expression in all the embryonic territories was observed. This could suggest that novel mesodermal lineages diverged from an ancestral mesoderm through only a few regulatory changes that drove major changes in downstream gene expression and embryonic morphology^7,8^.

Insight on the genome-wide changes in developmental gene expression can be gained from comparative transcriptome studies of related species^16–21^. We previously investigated different aspects of interspecies conservation of gene expression between *P*. *lividus* and the pacific sea urchin, *Strongylocentrotus purpuratus* (*S*. *purpuratus*)^18,19^. These species diverged from their common ancestor about 40 million years ago and have a highly similar embryonic morphology (Fig. 1A). We observed high conservation of gene expression dynamics of both genes that regulate developmental processes and of housekeeping genes (*e*.*g*., ribosomal and mitochondrial genes)^19^. Yet, the interspecies correlations of the expression levels of these two sets of genes show distinct patterns.

Before we describe the observed patterns, we would like to describe the structure and biological meaning of the two dimensional correlation matrix between different developmental stages in two species^19,22,23^. In such a matrix, the columns correspond to the developmental stages in one species and the rows to the developmental stages in the other species. The diagonal elements of the matrix, have the same column and row indices, *ii*, and show the correlations in gene expression between morphologically equivalent developmental stages in the two species. The off-diagonal elements of the matrix, have distinct column and row indices, *ij*, *i* *≠* *j*, and present the correlations in gene expression between morphologically distinct developmental stages. With increasing evolutionary distance, it becomes harder to properly identify morphologically equivalent stages. However, usually it is still possible for animals in the same phylum and straightforward for the comparison of the mentioned two sea urchin species^19^.

Thus, for *P*. *lividus* and *S*. *purpuratus*, the interspecies correlations of the expression levels of developmental genes are high between morphologically similar stages and decrease sharply between diverse developmental stages, resulting with highly diagonal correlation matrices^19^. The correlations peak at mid-development, at the onset of gastrulation, in agreement with the hourglass model of developmental conservation^19–21,24,25^. Conversely, the interspecies correlations of housekeeping gene expression increase with developmental time and are high between all post-hatching time points, resulting with distinct off-diagonal elements of the correlation matrix^19^. This indicates that the expression levels of housekeeping genes are correlated throughout the developmental of the two species, irrespective of specific morphological stages; which is reasonable, as these genes are expressed in all the cells throughout development. This could suggest that when the off-diagonal elements of the interspecies correlation matrix are of the same scale as the diagonal, the correlation in gene expression might reflect cellular and not development constraints.

Apparent differences in the conservation patterns between developmental and housekeeping genes were observed in other studies of closely related species^24^ and were a reason to exclude housekeeping genes from comparative studies of developing embryos^25^. Yet, embryogenesis progression depends on the dynamic expression of housekeeping genes and therefore we believe that these genes and their contribution to the overall correlation patterns should be considered^19^. Here we aim to decipher how the interspecies correlations in gene-expression change with increasing evolutionary distance. To this end, we generated and analyzed *de-novo* developmental quantitative transcriptomes of the sea star, *P*. *miniata*, and compared them with the published developmental transcriptomes of *P*. *lividus*^19^ and *S*. *purpuratus*^26^ at equivalent developmental stages (Fig. 1B). Our studies illuminate how the correlations of gene expression levels change with increasing evolutionary distance in both correlation strength and correlation matrix diagonality for different functional classes and embryonic territories.

## Results

### Developmental transcriptomes of the sea star, *P*. *miniata*

To study the transcriptional profiles of the sea star species, *P*. *miniata*, from fertilized egg to late gastrula stage and compare them to those in the sea urchin species, *S*. *purpuratus* and *P*. *lividus* we collected embryos at eight developmental stages matching to those studied the sea urchin species^19,26,27^ (Fig. 1B). Details on reference transcriptome assembly, quantification and annotations are provided in the Materials and Methods section. Quantification and annotations of all identified *P*. *miniata* transcripts are provided in Dataset S2. The temporal expression profiles of selected regulatory genes show variable levels of similarity between *P*. *miniata* (RNA-seq, current study) and the sea urchin species, *S*. *purpuratus* (nanostring^28^) and *P*. *lividus* (RNA-seq^19^, Fig. 1C). The temporal expression of some genes seem to be highly conserved (*e*.*g*., *foxa* and *six3*) while others show some divergence (*e*.*g*., *nodal* and *bra*) in agreement with our previous study (Fig. 1C)^14^.

### Identification of 1:1:1 homologous gene set and quantitative data website

**Figure 2:**
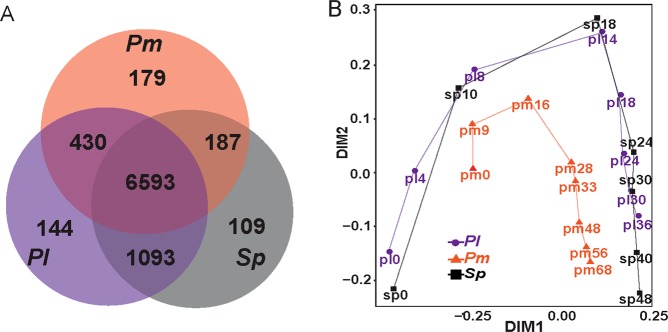
Venn diagram and NMDS analysis of 1:1:1 homologous genes. (**A**) Venn diagram showing the number of 1:1:1 homologous genes expressed in all three species, in two of the species or only in one species. (**B**) First two principal components of expression variation (NMDS) between different developmental time points in *P*. *miniata* (orange), *P*. *lividus* (purple) and *S*. *purpuratus* (black).

To compare developmental gene expression between the sea star, *P*. *miniata*, and the two sea urchins, *P*. *lividus* and *S*. *purpuratus* we identified 8735 1:1:1 putative homologous genes, as described in the Materials and Methods section. Quantification and annotations of all these homologous genes in *P*. *miniata*, *P*. *lividus* and in *S*. *purpuratus* are provided in Dataset S3 (based on^19^ for *P*. *lividus* and on^26,27^ *for S*. *purpuratus*). Within this set, 6593 genes were expressed in the three species, 1093 genes are expressed only in the two sea urchin species, 187 genes were expressed only in the sea star, *P*. *miniata*, and the sea urchin, *S*. *purpuratus*, and 430 genes are expressed only in *P*. *miniata* and *P*. *lividus* (Fig. 2A). We looked for enrichment of gene ontology (GO) terms within these different gene sets but did not identify enrichment of specific developmental processes (GOseq^29^ with *S*. *purpuratus* annotations, Fig. S1 and Dataset S4).

We uploaded the data of the 1:1:1 homologous genes expressed in the three species to Echinobase where they are available through gene search at www.echinobase.org/shiny/quantdevPm ^30^. In this Shiny web application^31^, genes can be searched either by their name or by their *P*. *miniata*, *S*. *purpuratus* or *P*. *lividus* transcript identification number. The application returns our quantitative measurements of gene expression for each stage of *P*. *miniata*, plots of transcript expression level, mRNA sequences, a link to the corresponding records in Echinobase^32^ and links to the loci in the *S*. *purpuratus* and *P*. *miniata* genome browsers. We hope that this resource will be useful for the community. Further analyses of the 6593 1:1:1 homologous genes expressed in all three species are described below.

### Main variations in gene expression profiles are due to developmental progression and evolutionary distance

To identify the general trends of gene expression over developmental time in the three species, we performed a non-metric multidimensional scaling analysis (NMDS, Fig. 2B). *S*. *purpuratus* RNA-seq data does not include the early development time point equivalent to the *P*. *miniata* 9 hours post fertilization (hpf) and *P*. *lividus* 4 hpf^26^. The NMDS maps the developmental trajectories of the two sea urchin species closely to each other (purple and black tracks), while the sea star samples are relatively distinct (orange track). This is in agreement with the phylogenetic relationships between the three species (Fig. 1A). The developmental progression in all three species is along a similar trajectory in the NMDS two-dimensional space, possibly reflecting the resemblance in the overall morphology between these three echinoderm species. Overall, the NMDS indicates that major sources of variation in these data sets are developmental progression and evolutionary distance, in agreement with previous comparative analysis of the transcriptomes of three other echinoderm species^17^.

### Interspecies correlations decrease and become less diagonal with evolutionary distance

**Figure 3:**
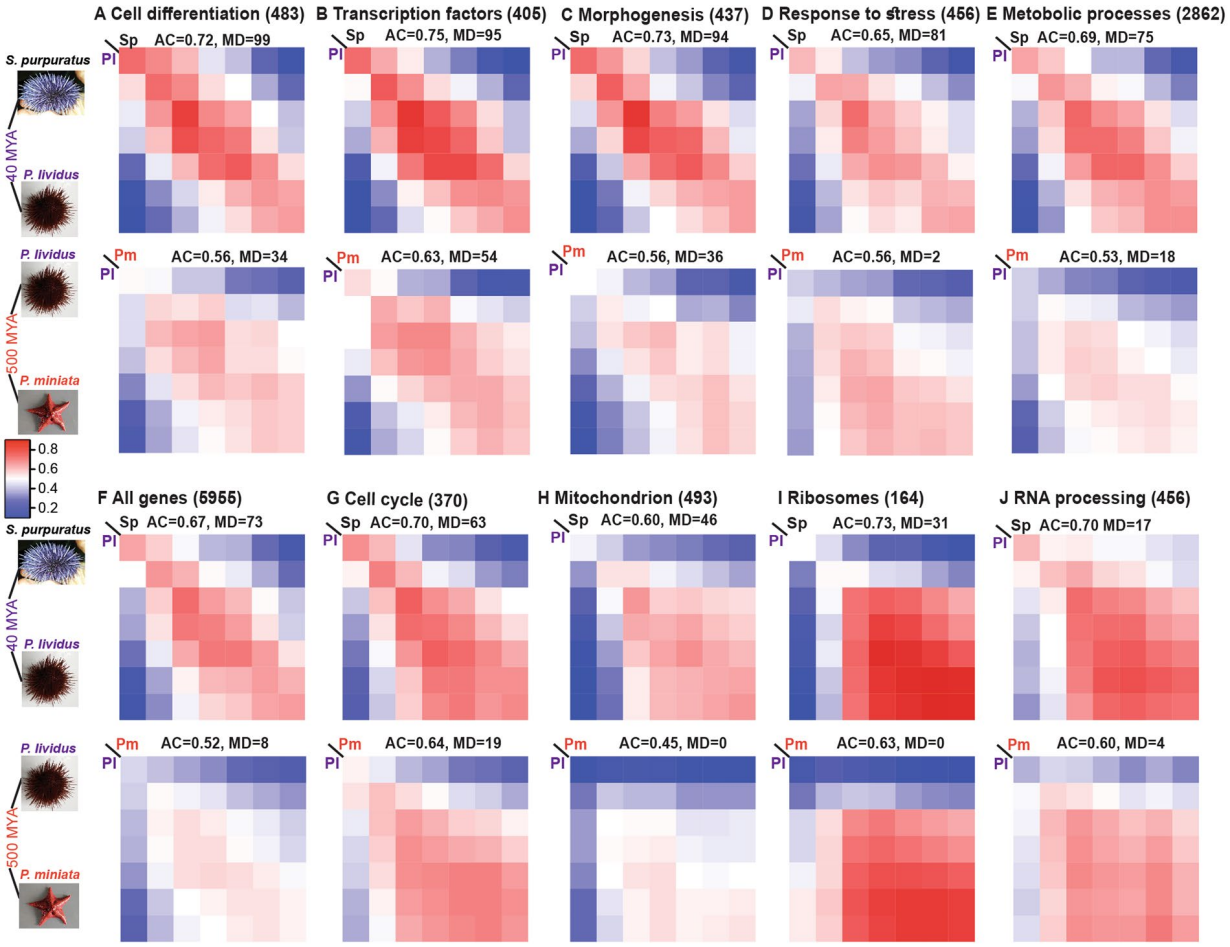
Interspecies Pearson correlations for different GO terms, ordered by the level of matrix diagonality (MD) of *Pl-Sp* matrices. In each panel, from (**A**–**J**) we present the Pearson correlation of the expression levels of genes with specific GO term between different developmental stages in two species. Upper matrix in each panel shows the Pearson correlation between the two sea urchins (*P*. *lividus* and *S*. *purpuratus*) and the bottom matrix is the Pearson correlation between the sea star, *P*. *miniata* and the sea urchin *P*. *lividus*. These matrices include the seven developmental points that have RNA-seq data in all species (Fig. 1B, excluding 9 hpf in *P*. *miniata* and 4 hpf in *P*. *lividus*). In each panel we indicate the GO term tested, the number of genes in each set, the average correlation strength in the diagonal (AC) and the matrix diagonality (MD), see text for explanation. Linear color scale of Pearson correlations 0–1 is identical for all graphs and given at the middle of the figure. (**F**) Shows the Pearson interspecies correlation for all 1:1:1 genes.

We wanted to investigate how the pattern of the interspecies correlations of gene expression changes with evolutionary distance for different classes of genes. To be able to compare the interspecies correlations between the three species we included only time points that had data for all species. Explicitly, we excluded *P*. *lividus* 4 hpf and *P*. *miniata* 9 hpf that do not have an equivalent time point in the *S*. *purpuratus* data. We calculated the Pearson correlations of gene expression levels between *P*. *lividus* and *S*. *purpuratus*, between *P*. *lividus* and *P*. *miniata* (Fig. 3) and between *S*. *purpuratus* and *P*. *miniata* (Fig. S2). We did that for subsets of homologous genes with specific GO terms that describe developmental, housekeeping, response or metabolic function. As expected, the strength of the interspecies correlation decrease at increasing evolutionary distance, for all classes of genes (Figs 3 and S2). Explicitly, the correlations are stronger between the two sea urchin than between the sea urchins and the sea star (Figs 3 and S2). This is in agreement with our previous observation that the expression kinetics and initiation times of key developmental genes show higher conservation between the two sea urchins than between the sea urchins and the sea star^14^. Additionally, the correlations between morphologically equivalent stages become more similar to the correlations between distinct stages, that is, the *Pm-Pl* and *Pm-Sp* matrices are less diagonal compared to the *Pl-Sp* matrices (Figs 3 and S2).

For a better assessment of the correlation patterns we sought to quantify these two distinct properties: the correlation strength between equivalent developmental stages and the diagonality of the matrix. As a measure of the correlation strength between morphologically similar time points, we defined the parameter AC = Average Correlation, which is the average of the diagonal elements of the correlation matrix (the matrix elements that have the same column and row indices and show the correlations in gene expression between morphologically equivalent stages in the two species). That is, AC = 1 corresponds to perfect correlation throughout all equivalent developmental stages and AC = 0 corresponds to no correlation. Evidently, AC is not a measure of the diagonality of the matrix as it doesn’t consider the off-diagonal elements (the matrix elements that have distinct column and row indices and show the correlations in gene expression between morphologically distinct stages in the two species). To quantify the diagonality of the correlation matrices we used a statistical test we developed before to assess whether the interspecies correlation pattern is significantly close to a diagonal matrix (where all the diagonal elements are 1 and the off-diagonal are 0)^19^. Briefly, the parameter, matrix diagonality (MD), indicates how many times within 100 subsamples of the tested set of genes, the interspecies correlation matrix was significantly close to a diagonal matrix compared to a random matrix. Hence, MD = 100 is the highest diagonality score and 0 is the lowest (see Materials and Methods section and^19^ for explanation, and Dataset S5 for results. MD is equivalent to the parameter ‘count significant’, or CS, in^19^). Importantly, AC will be high for any matrix that has high diagonal elements, but MD will be high only when the off-diagonal matrix elements are much lower than the diagonal.

We measured these parameters for the three comparisons, *Pm-Pl*, *Pl-Sp* and *Pm-Sp* and observed similar trends for both sea urchin–sea star comparisons (*Pm-Pl* and *Pm-Sp*, see Figs 3 and S2). Therefore, for simplicity of the discussion, in the rest of the paper we focus on the quantification results of the *Pm-Pl* and *Pl-Sp* matrices. The reason for preferring *Pm-Pl* over *Pm-Sp* is that the RNA-sequencing of *P*. *miniata* and *P*. *lividus* were conducted by us in the same facility and included three biological replicates for each time point (see Materials and Methods for *P*. *miniata* and^19^ for *P*. *lividus*).

In Fig. 3, we ordered the correlation matrices according to their MD value in *Sp-Pl*, from the most diagonal (Cell differentiation, MD = 99) to the least (RNA processing, MD = 17). This ordering clearly demonstrates the high diagonality of the developmental genes (Fig. 3A–C) vs. the block patterns of the housekeeping genes (Fig. 3G–J) and how the diagonality of the correlation pattern of response to stress genes, metabolic genes and all genes combined are in between (Fig. 3D–F). Both the average correlation and the matrix diagonality are lower between the sea urchin and the sea star than between the two sea urchins, that is, both parameters decrease with evolutionary distance (compare bottom to upper panels in Fig. 3). The reduction in AC indicates that the correlation strength between morphologically equivalent stages decreases, which could be due to drift, environmental adaptation, or morphological divergence at increasing evolutionary distance. The reduction in matrix diagonality means that the correlation in gene expression between morphologically similar developmental stages becomes of the same order as the correlation between distinct developmental stages. Thus, for an increasing number of gene sets at large evolutionary distance the correlation of gene expression levels between different stages become similar, regardless of morphological similarity. This could suggest that the morphological constraints are less dominant in controlling the conservation of gene expression compared to other cellular or metabolic constraints, with increasing evolutionary distance.

### The interspecies correlations vary between different embryonic territories

Our analysis is based on RNA-seq on whole embryos, yet we wanted to see if gene sets enriched in specific cell populations show a difference in their interspecies correlation pattern. In a previous work conducted in the sea urchin *S*. *purpuratus* embryos, the cells of six distinct embryonic territories were isolated based on cell-specific GFP reporter expression. Gene expression levels in the isolated cells were studied and compared to gene expression levels in the rest of the embryo by RNA-seq^33^. This analysis identified genes whose expression is enriched in specific cell populations at the developmental time when the isolation was done^33^. We calculated the Pearson interspecies correlations for the subsets of genes enriched in each of the six sea urchin embryonic territories, between the two sea urchins and between the sea urchins and the sea star (Figs S2 and S3).

**Figure 4:**
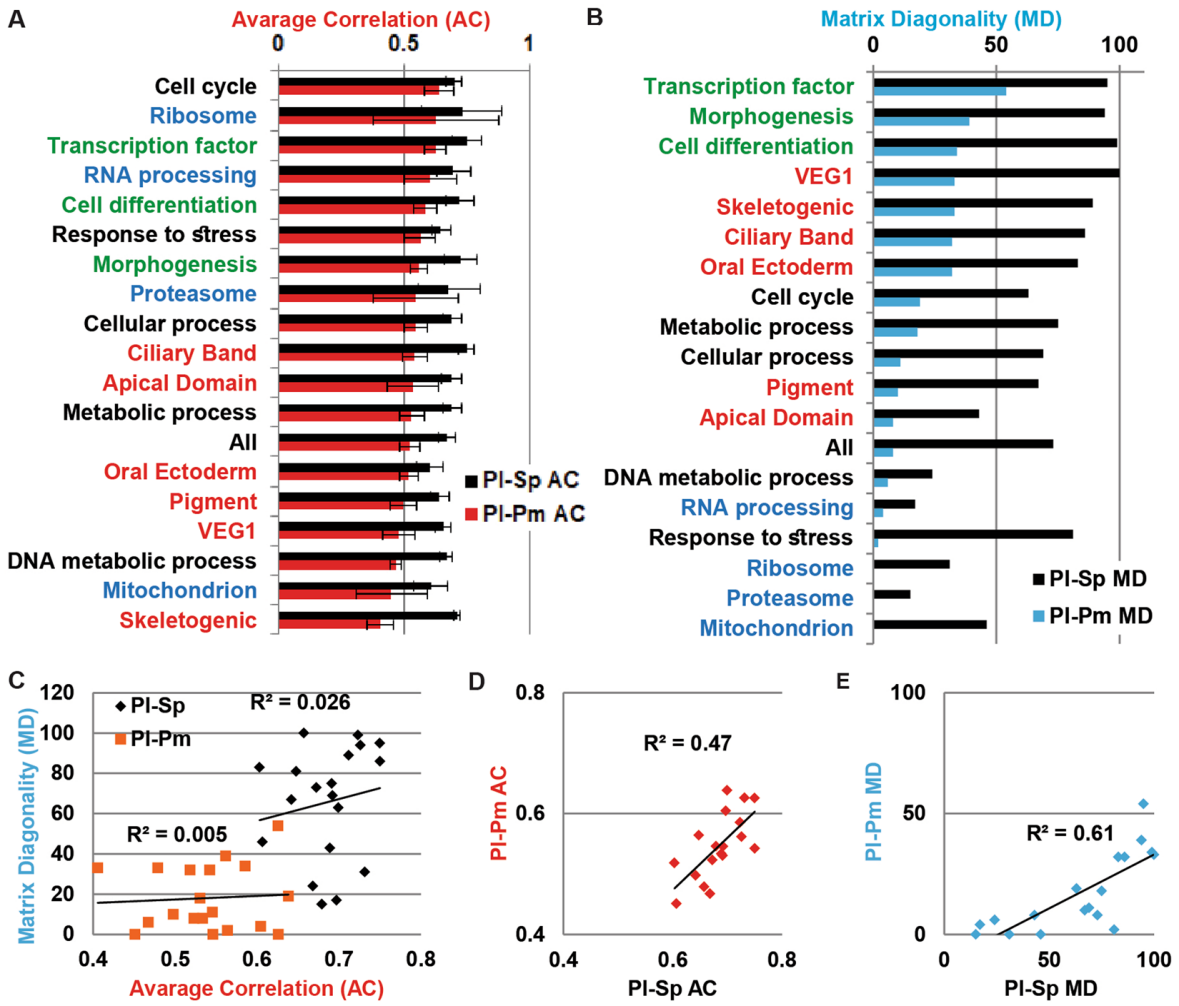
The average correlation strength and the matrix diagonality are independent parameters that reflect different properties of expression conservation. (**A**) The average correlation strength (AC) between *Pl-Sp* (black bars) and *Pl-Pm* (red bars) in receding order of *Pl-Pm* correlation strength. Error bars indicate standard deviation of the correlation strength along the matrix diagonal. (**B**) Matrix diagonality (MD) of the interspecies correlations between *Pl-Sp* (black bars) and *Pl-Pm* (cyan bars) in receding order of *Pl-Pm* matrix diagonality. In (**A**,**B**) the different gene sets are colored by the following key: developmental GO terms in green, housekeeping GO terms in blue, lineage specific genes in red and all the other sets in black. (**C**) The matrix diagonality changes independently of the average correlation for both *Pl-Sp* (black dots) and *Pl-Pm* (orange dots). (**D**) The interspecies average correlation between *S*. *purpuratus* and *P*. *lividus* corresponds to the interspecies average correlation between *P*. *lividus* and *P*. *miniata* (R pearson = 0.68). (**E**) The matrix diagonality of the interspecies correlations between *S*. *purpuratus* and *P*. *lividus* relates to the matrix diagonality between *P*. *lividus* and *P*. *miniata* (R Pearson = 0.78). In (**D**,**E**) we didn’t include the skeletogenic lineage data point as it is an outlier.

Both the matrix diagonality and average correlation strength vary between the different embryonic territories and decrease with evolutionary distance. Interestingly, the genes enriched in sea urchin pigment cells, a lineage that is lacking in the sea star, show similar *Pl-Pm* average correlation strength compared to the matrices of the embryonic territories that are common to the sea urchins and the sea star (*Pl-Sp* AC = 0.64 and *Pl-Pm* AC = 0.5, Fig. S3B). As mentioned above, the regulatory state in the mesoderm of the sea star and the sea urchin are quite similar^7^. Possibly, there is also similarity in the downstream genes active in sea star blastocoelar cells and sea urchin pigment cells, as both lineages function as immune cells in the sea urchin^34^. On the other hand, the sharpest decrease in *Pl-Pm* average correlation strength compared to *Pl-Sp* is for the genes enriched in the sea urchin skeletogenic cells, another lineage that is absent in the sea star (*Pl-Sp* AC = 0.71 while *Pl-Pm* AC = 0.4, Fig. S3C). Interestingly, the *Pl-Pm* matrix diagonality of these genes is comparable to the *Pl-Pm* matrix diagonality of the common lineages (*Pl-Pm* MD = 33, Fig. 4C). It is important to note that key sea urchin skeletogenic matrix proteins were not found in the sea star skeleton^35^ and the genes encoding them were not found in the sea star genome. Therefore, these key skeletogenic genes are missing from our 1:1:1 homologous genes that include only genes that are common to the three species. Thus, we are probably underestimating the differences in skeletogenic and mesodermal gene expression between the sea urchin and the sea star, which might explain the relatively high diagonality of the skeletogenic gene correlation matrix. Overall, genes enriched in different cells populations differ in their correlation patterns even between the closely related sea urchins and show distinct differences in the correlation pattern with evolutionary distance.

### Matrix diagonality and correlation strength describe different properties of gene expression conservation

The average correlation strength and the matrix diagonality seem to change independently from each other for different gene functions and cell populations (Figs 3, S2 and S3). To further investigate that we plot, separately, the average correlation and the matrix diagonality for different GO terms and cell populations, in decreasing strength in *Pl-Pm* and observed two distinct orders (Fig. 4A,B). Ordering by matrix diagonality separates between genes with developmental GO terms or specific cell populations (high diagonality) and genes with housekeeping GO terms (low diagonality, Fig. 4B). Ordering by the average correlation does not show this separation (Fig. 4A). Moreover, the matrix diagonality and the average correlation strength change independently of each other in both *Pl-Sp* and *Pl-Pm* correlation matrices (Fig. 4C). On the other hand, the average correlations in *Pl-Sp* matrices seem to correspond to the average correlation in *Pl-Pm* (Fig. 4D), and the same is true for the matrix diagonality (Fig. 4E).

**Figure 5:**
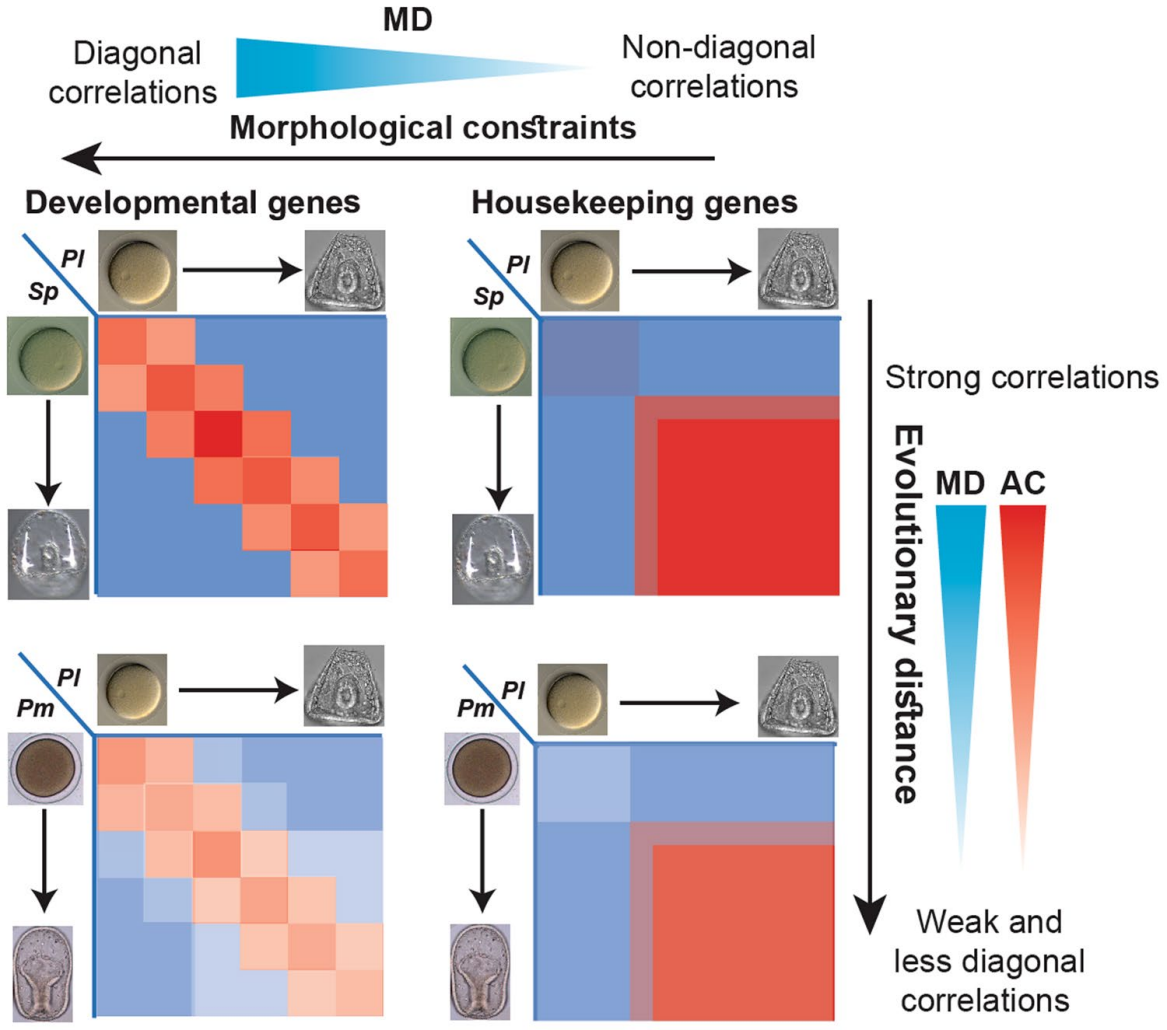
The correlation matrix diagonality, MD, reflects how the dominance between cellular and developmental constraints changes with evolutionary distance. Illustration of typical interspecies correlation matrices of developmental control genes and housekeeping genes between closely related and further diverged species (upper and lower panels, respectively). With increasing evolutionary distance, that is, between the sea urchin and the sea star, the average correlation and the diagonality decrease for all gene sets but the diagonality of developmental control genes is least affected and they still maintain the hourglass pattern (left panels). On the other hand, the interspecies correlation matrices of housekeeping genes are strong and non-diagonal even between the two sea urchins and remain non-diagonal between the sea urchin and the sea star (right panels).

These different behaviors of the average correlation and the matrix diagonality could suggest that that these two parameters describe different properties of the conservation in gene expression, as we propose in Fig. 5. The average correlation strength seems to reflect the conservation in gene expression levels: the higher it is, the more the gene-set is constrained against expression change. The matrix diagonality on the other hand, seems to reflect the link between the expression of a gene-set and morphological constraints on this set: the more diagonal is the correlation pattern of a gene set, the more dominant is the morphological constraint on the expression conservation within the set (Fig. 5). For example, the correlation matrices of transcription factors and cell differentiation genes show strong correlations and high diagonality even between the sea urchin and the sea star (Relatively high AC and MD, Figs 3A,B and 5). Conversely, ribosomal gene expression is highly conserved (high AC) but this conservation is not related to morphological conservation (Low MD, Figs 3I and 5). Overall, the average correlation strength and matrix diagonality seem to carry complementary information about the relationship between the conservation of gene expression and morphological conservation at varying evolutionary distances (Fig. 5).

## Discussion

In this paper we generated the developmental transcriptomes of the pacific sea star, *P*. *miniata*, and studied them in comparison with the published developmental transcriptomes of two sea urchin species, *P*. *lividus*^19^ and *S*. *purpuratus*^26^. We generated a web application where the *P*. *miniata* time courses and sequences can be publicly viewed to facilitate the common use of this data^30^. We studied the interspecies correlation patterns of different gene sets including, housekeeping, developmental, response and metabolic genes (Figs 3 and S2), as well as genes that are enriched in specific cell populations in the sea urchin embryo (Figs S2 and S3). We defined two parameters that describe different properties of the conservation strength: the average correlation strength in the diagonal, AC, and the matrix diagonality, MD. We noticed that these parameters vary independently between different functional groups and cell populations and decrease with evolutionary distance, possibly reflecting different constraints on gene expression (Fig. 4). The correlation strength seems to indicate the evolutionary constraint on gene expression level while the matrix diagonality seems to reflect the dominance of morphological constraints on the conservation of gene expression within a gene set (Fig. 5). As we suggested previously, parallel embryonic transcriptional programs might be responsible for different aspects of embryo development and evolve under distinct constraint^19^, as can be inferred from analyzing these two parameters.

Previous studies have shown that housekeeping genes and tissue specific genes have different chromatin structures^36^ and distinct core promoters^37^. Furthermore, Enhancers of developmental genes were shown to be de-methylated during the vertebrates’ phylotypic period, suggesting another unique epigenetic regulation of this set of genes^38^. These epigenetic and *cis*-regulatory differences could underlie the separation of the regulation of developmental and housekeeping gene expression, leading to dissimilar evolutionary conservation patterns of these two functionally distinct gene sets.

We observed a clear reduction of the matrix diagonality with increasing evolutionary distance for all sets of genes (Figs 3–5, S2 and S3). This is in agreement with the morphological and evolutionary divergence between the sea urchin and the sea star. However, it is important to note that in the comparisons conducted in this work the phylogenetic distance and morphological divergence are interconnected: the two sea urchins are phylogenetically close and morphologically similar, while the sea urchins and the sea star are phylogenetically distant and morphologically divergent. Thus, in these comparisons it is impossible to disentangle the effects of phylogenetic distance and morphological divergence. It would be interesting to study the correlation strength and matrix diagonality between phylogenetically close but morphologically diverged species. Possible example could be sea urchin species in the genus Heliocidaris that rapidly transitioned from feeding to nonfeeding larval development and therefore show significant morphological divergence within a very short phylogenetic distance^17^. We hypothesize that in this kind of comparison, the average correlation strength will associate with the phylogenetic distance and will be strong within the closely related and morphologically diverged Heliocidaris species. On the other hand, the matrix diagonality will correlate with the morphological similarity and will show higher diagonality between phylogenetically distance species that have similar morphology. Thus, we expect these two parameters to show opposite trends when the phylogenetic distance and morphological similarities are distinct.

A recent study has shown that the correlation matrices for all homologous genes between 10 different phyla are strictly not-diagonal^22^. At this large evolutionary distance and extreme morphological divergence, the dominant constraint is probably the cellular requirements for differential transcript abundance, which are unrelated to morphological similarity. Therefore the opposite hourglass pattern found for the diagonal elements of the interspecies correlation matrices between different phyla might not be indicative for morphological divergence at the phylotypic stage^22^. Overall, to better estimate the relationship between the conservation of gene expression levels to morphological conservation, both correlation strength and correlation matrix diagonality should be assessed and the focus should be on developmental control genes.

## Materials and Methods

### Sea star embryo cultures and RNA extraction

Adult *P*. *miniata* sea stars were obtained in Long Beach, California, from Peter Halmay. Embryos were cultured at 15 °C in artificial sea water. Total RNA was extracted using Qiagen mini RNeasy kit from thousands of embryos. RNA samples were collected from eight embryonic stages, from fertilized egg to late gastrula stage (Fig. 1B). For each embryonic stage, three biological replicates from three different sets of parents were processed, except for the last time point for which only two biological replicates were sampled (23 samples in all). To match *P*. *miniata* time points to those of the published *S*. *purpuratus* and *P*. *lividus* transcriptomes^19,27^ we used the linear ratio between the developmental rates of these species found in^14,39^. The corresponding time points in both species are presented in Fig. 1B. The time points, 9hpf in *P*. *miniata* and 4hpf in *P*. *lividus* do not have a comparable time point in *S*. *purpuratus* data. RNA quantity was measured by nanodrop and quality was verified using bioanalyzer.

### Transcriptome assembly, annotations and quantification

#### RNA-Seq preparation

The 23 *P*. *miniata* samples that we analyze here were processed together with 24 *P*. *lividus* samples that we described in^19^. Library preparation was done at the Israel National Center for Personalized Medicine (INCPM) as following. polyA fraction (mRNA) was purified from 500 ng of total RNA per developmental time point followed by fragmentation and generation of double stranded cDNA. Then, end repair, A base addition, adapter ligation and PCR amplification steps were performed. Libraries were evaluated by Qubit and TapeStation. Sequencing libraries were constructed with barcodes to allow multiplexing of 47 samples. On average, 20 million single-end 60-bp reads were sequenced per sample on Illumina HiSeq 2500 V4 using four lanes. Exact number of reads for each sample is provided in Dataset S1.

#### RNA-Seq datasets used

Three datasets were used for transcripts quantification: (1) newly sequenced *P*. *miniata* Single End (SE) reads of 23 transcriptome samples generated as explained above; (2) publicly available SE reads of *P*. *lividus* transcriptome samples from eight developmental stages (NCBI project PRJNA375820)^19^; (3) publicly available Paired End (PE) reads of seven *S*. *purpuratus* transcriptome samples (NCBI project PRJNA81157)^27^. For generating reference transcriptome for *P*. *miniata* we used PE reads of *P*. *miniata* from different developmental stages, testis and ovaries, accessions: SRR6054712, SRR5986254, SRR2454338, SRR1138705, SRR573705-SRR573710 and SRR573675.

#### RNA-seq quality filtering

RNA-Seq reads from the above datasets were adapter-trimmed using cutadapt 1.15 (https://cutadapt.readthedocs.io), then low-quality regions were removed with Trimmomatic 0.3^40^, and further visually inspected in fastq-screen (www.bioinformatics.babraham.ac.uk).

#### *P*. *miniata* transcriptome assembly

While *P*. *miniata* genome based gene predictions are available for genome assembly v.2 (echinobase.org/Echinobase), many gene sequences are fragmented or duplicated within the genome, and therefore we decided to generate a reference transcriptome *de-novo*. Accordingly, *P*. *miniata* RNA-Seq reads, based on the available PE and SE data, were assembled using Trinity 2.4^41,42^ with the same Trinity parameters as we used for *P*. *lividus* in^19^. Trinity produced 1,610,829 contigs (the Trinity equivalent of transcripts), within 679,326 Trinity gene groups. Transciptome completeness was tested using BUSCO^43^, and by comparing the contigs to genome-based protein annotations *P*. *miniata* v.2.0 and *S*. *purpuratus* v.3.1 (WHL22), (echinobase.org/Echinobase).

#### *P*. *miniata* data availability

Illumina short read sequences generated in this study were submitted to the NCBI Sequence Read Archive (SRA) (http://www.ncbi.nlm.nih.gov/sra), under bio-project PRJNA522463 (http://www.ncbi.nlm.nih.gov/bioproject/522463). Fastq read accessions: SRR8580044–SRR8580066, assembled *P*. *miniara* transcriptome accession: SAMN10967027.

#### Transcriptome homology

We searched for homologous genes in the *P*. *miniata* transcriptome, *P*. *lividus* transcriptome, *S*. *purpuratus* genome. Since the *S*. *purpuratus* genome-based gene predictions dataset currently includes the most non-redundant and complete data among the three datasets, it was used as a reference dataset. Accordingly, the largest isoforms of new *P*. *miniata* transcriptome (see below), and the publicly available *P*. *lividus* transcriptome^19^, were both compared to the *S*.*purpuratus* genome-based predicted protein annotations (Echinobase v.3.1), using CRB-BLAST^44^. CRB-BLAST reports relationships of 1:1 (Reciprocal hits), and 2:1 matches (when Blastx and tBlastn results are not reciprocal), where only matches with e-values below a conditional threshold are reported. From the 2:1 cases, we selected the query-target pair with the lowest e-value. Using CRB-BLAST, 11,291 and 12,720 *P*. *miniata* and *P*. *lividus* Trinity genes, were identified as homologous to *S*. *purpuratus* proteins, respectively. We considered *P*. *miniata* and *P*. *lividus* query genes that share the same *S*. *purpuratus* target gene, as homologous. As the gene expression analysis shows (see next sections), 8,735 homologous genes are expressed in at least one of the three tested species during development, and 6,593 are expressed in all the three.

#### Gene-level transcripts abundance

For *P*. *miniata* and *P*. *lividus* transcriptomes, transcripts abundance was estimated using kallisto-0.44.0^45^, and a further quantification at the gene-level, and read-count level, was done using tximport^46^ on R3.4.2. Expression analysis at read-count level was conducted at gene-level in Deseq2^47^. *S*. *purpuratus* PE reads, of 7 developmental samples were mapped to the *S*. *Purpuratus 3*.1 genome assembly, using STAR v2.4.2a^48^, quantitated in Htseq-count v2.7^49^, and analyzed in Deseq2 at read-count level. For *P*. *miniata* data, only contigs mapped to the Echinobase v.2 *P*. *miniata* proteins were considered. Prior to DEseq2 read count standard normalization and expression analysis, genes with <1 CPM (Count Per Million) were removed. Overall, most input reads were mapped and quantified, as further detailed in Dataset S1. Samples were clustered using Non-metric multi-dimentional scaling (NMDS) ordination in Vegan (https://cran.r-project.org/web/packages/vegan/index.html), based on log10 transformed FPKM values, and Bray-Curtis distances between samples. Since the NMDS results indicate that all *P*. *miniata* and *P*. *lividus* samples are affected by the ‘batch’ factor (see Dataset S1), we removed the estimated effect of this factor on FPKMs (Fragment per Kilobase Million) values using “removeBatchEffect” function in EdgeR^50^, in order to obtain corrected FPKM values. Quantification and annotations of 34,307 identified *P*. *miniata* transcripts with FPKM >3 in at least one time point, are provided in Dataset S2. NMDS analysis of the biological replicates of all time points in *P*. *miniata* show that similar time points at different biological replicate map together indicating high reproducibility of our gene expression analysis (Fig. S4). Comparison between our RNA-seq quantification of gene expression and previous QPCR quantification^14^ for a subset of genes show high agreement between the two measurements (Fig. S5). Quantification and annotations of the 8,735 identified *P*. *miniata*, *P*. *lividus and S*. *purpuratus* 1:1:1 homologous genes are provided in Dataset S3 and are publicly available through gene search in Echinobase at www.echinobase.org/shiny/quantdevPm.

#### Gene Ontology functional enrichment analysis

Functional enrichment analysis was conducted using TopGo in R3.4.2 (bioconductor.org). A custom GOseq GO database was built using the publicly available Blast2Go *S*. *purpuratus v3*.*1* (WHL22) version (http://www.echinobase.org/Echinobase/rnaseq/download/blast2go-whl.annot.txt.gz).

#### Pearson correlations for subsets of genes

The interspecies Pearson correlations for different sets of genes presented in Figs 3, S2 and S3 were calculated using R3.4.2. For the analysis in Fig. S3, we selected genes that have 1:1:1 homologs in *P*. *miniata* and *P*. *lividus* to the *S*. *purpuratus* genes that are enriched in a specific *S*. *purpuratus* cell population with p-value < 0.05, based on^33^.

#### Cross species analysis of matrix diagonality (MD)

We used a statistical test described in detail in^19^. Shorty, the main goal of this procedure is to test the probability that a set of homologous genes from two species, *S*_1_ and *S*_2_, show the most similar expression patterns in equivalent developmental times, namely: is the interspecies correlation pattern significantly close to a diagonal matrix? Here, *S*_1_ and *S*_2_ represent *P*. *miniata* vs. *P*. *lividus*, or *P*. *lividus* vs. *S*. *purpuratus*. This test was conducted using all homologous genes, and for specific subsets of genes belonging to specific GO categories as well as for genes enriched in specific cell population^33^. Here, only samples from the *n* = 5 late embryonic stages were used, and the mean of FPKM values of all samples belonging to the same stages were taken. First, *n*_*s1*_ by *n*_*s2*_ matrix of Pearson correlations, *C*, was produced, where *n*_*s1*_ by *n*_*s2*_ are the number of time point measurements in each species (here *n*_*s1*_ = *n*_*s2*_ = 5). Each of the *C* matrix positions, *C*_*ij*_, represents a Pearson’s correlation value calculated based on *n*_*g*_ FPKM values, between stage *i* in one species and stage j in the other, where *n*_*g*_ is the count of homologous gene pairs tested. Next, the *C* matrix was compared to an “ideal” time-dependent correlation matrix *I*, in which a correlation of 1 exists along the diagonal line, and 0 in other positions, to obtain a diagonality measure, *d*. Overall, *n*_*g*_ = 50 genes were resampled *n*_*resamp*_ = 100 times, and for each resampling-iteration, a permutation test based on value *d* was applied using *n*_*perm*_ = 1000 permutations. Accordingly, each of the above *n*_*resamp*_ = 100 permutation tests indicate the probability that *S*_*1*_ and *S*_*2*_ show non-random diagonality, on a subset of n_g_ = 50 genes. Then, the count of permutation tests indicating a significant similarity to the ideal diagonal matrix, out of the *n*_*resamp*_ = 100 subsamples is used for estimating diagonality of the correlation matrix (matrix diagonality = MD). Our results are presented in Dataset S5 and within Figs 3, 4, S2 and S3.

## Supplementary information

Supplementary figures S1-S5

Dataset 1

Dataset 2

Dataset 3

Dataset 4

Dataset 4
