# Supplementary material for: Developmental transcriptomes of the sea star, Patiria miniata, illuminate how gene expression changes with evolutionary distance
Source: Sci Rep. 2019 Nov 7;9:16201. doi: 10.1038/s41598-019-52577-9 (PMC6838185; doi:10.1038/s41598-019-52577-9)
Supplement: Supplementary file 1 — Supplementary figures S1-S5 [file 41598_2019_52577_MOESM1_ESM.pdf]

Developmental transcriptomes of the sea star, *Patiria miniata*, illuminate how gene expression changes with evolutionary distance

Tsvia Gildor[1], Gregory Cary[3], Maya Lalzar[2], Veronica Hinman[3] and Smadar Ben-Tabou de-Leon[1,*]

[1]Department of Marine Biology, Leon H. Charney School of Marine Sciences, University of Haifa, Haifa 31905, Israel.

[2]Bionformatics Core Unit, University of Haifa, Haifa 31905, Israel.

[3]Departments of Biological Sciences and Computational Biology, Carnegie Mellon University Pittsburgh, PA 15213, USA

***Corresponding author** sben-tab@univ.haifa.ac.il

Supplementary figures

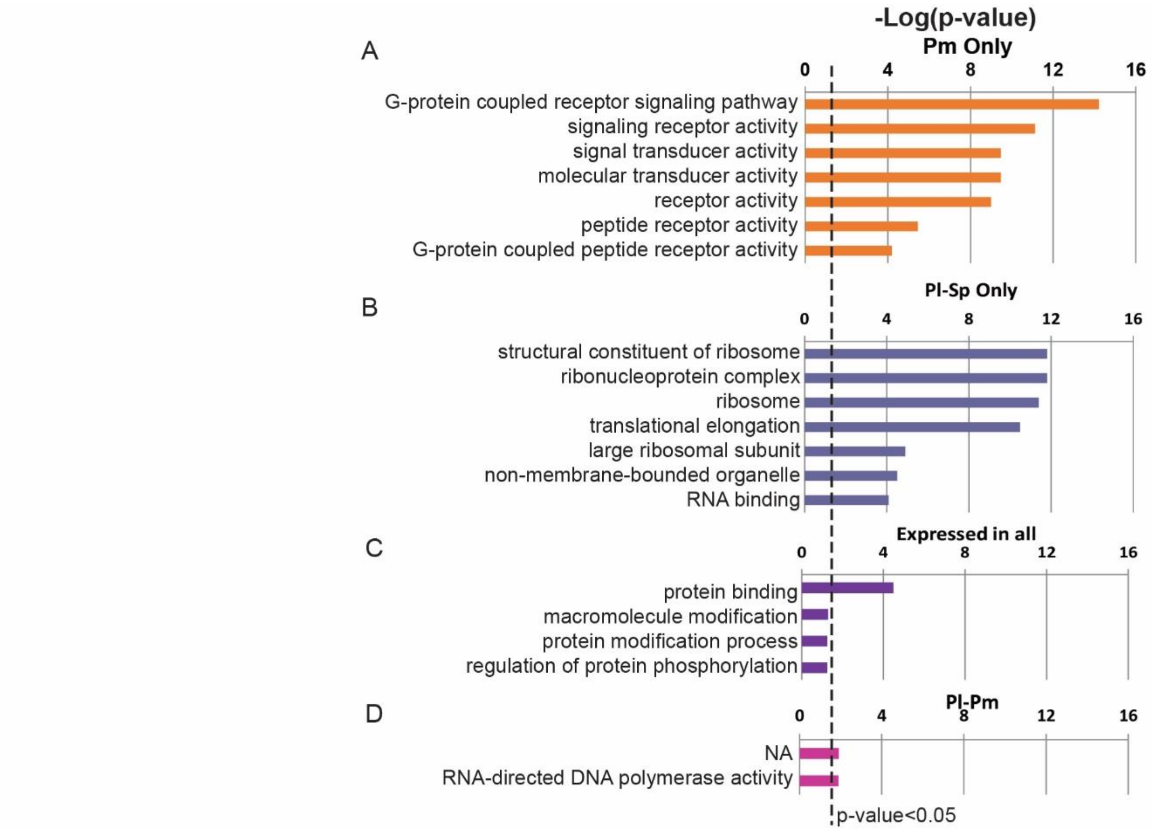

Figure S1 GO enrichment in the different expression groups. A, GO enrichment in genes expressed only in *P. miniata*, B, GO enrichment in gene expressed only in the two sea urchins, C, GO enrichment in genes that are expressed in the three species, D, GO enrichment in genes expressed in only in *P. lividus* and *P. miniata*.

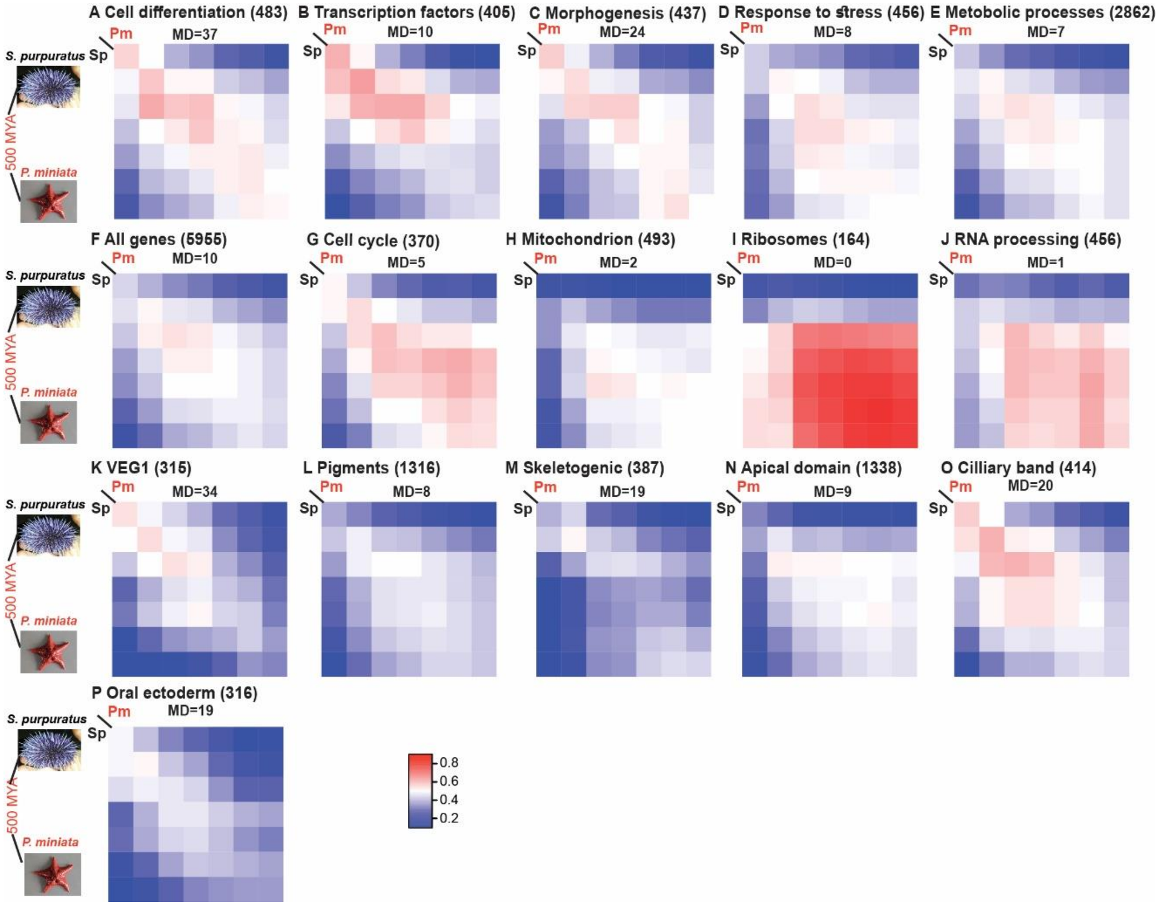

Figure S2 Interspecies Pearson correlations between *S. purpuratus* and *P. miniata* for different GO terms and genes expressed in specific cell lineages. In each panel, from A to P, we present the Pearson correlation of the expression levels of genes with specific GO term/cell lineage between different developmental stages is *S. purpuratus* and *P. miniata*. These matrices include the seven developmental points that have RNA-seq data in all species (Fig. 1B, excluding 9hpf in *P. miniata*). In each panel we indicate the GO term tested, the number of genes in each set and the matrix diagonality (MD), see text for explanation. Linear color scale of Pearson correlations 0-1 is identical for all graphs and given at the bottom of the figure. F, shows the Pearson interspecies correlation for all 1:1:1 genes.

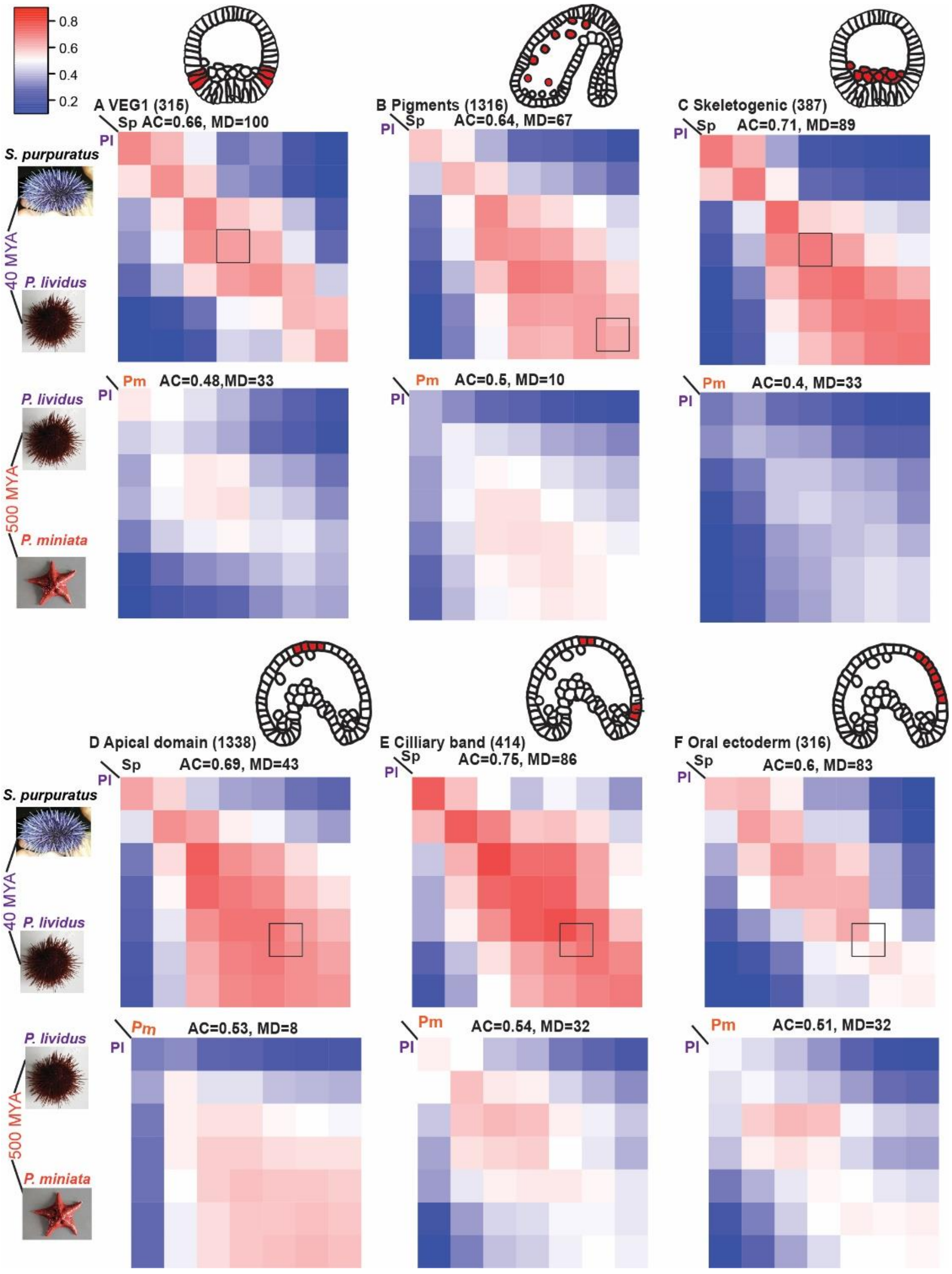

Figure S3 Interspecies Pearson correlations in gene expression for genes enriched in specific sea urchin cell populations. A-F, each panel shows the interspecies Pearson correlation between the developmental stages in the three species for genes that their expression enriched at specific time point in a particular embryonic territory in the sea urchin, *S. purpuratus* according to [1]. The embryonic territory at the time where the enrichment was studied are illustrated by the embryo diagrams above the relevant correlation pattern [1], the time point is also marked in a black square in each *Pl-Sp* panel, A, 24hpf; B, 45hpf; C, 24hpf; D-E, 35hpf. In each panel we indicate embryonic territory where these genes are enriched, the number of genes in each set, the average correlation strength in the diagonal (AC) and the matrix diagonality (MD), see text for explanation. Linear color scale of Pearson correlations 0-1 is given at the top-left of the figure and is identical for all graphs throughout the paper.

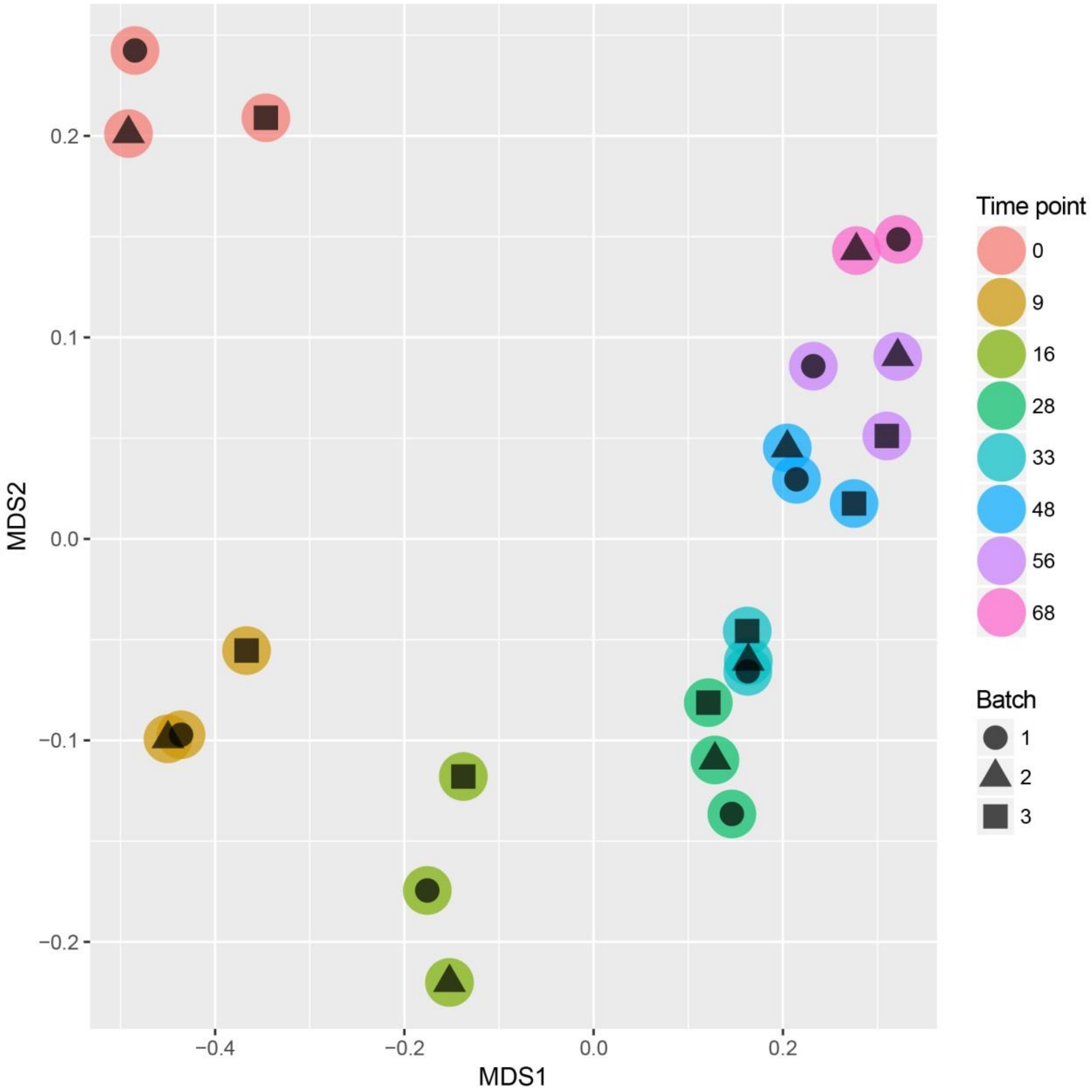

Figure S4 NMDS analysis of the sea star expression profiles for the three biological replicates show high reproducibility of gene expression. NMDS of individual *P. miniata* time points and biological replicates. The different batches are indicated as circles, triangles and squares and different developmental time points are indicated by color.

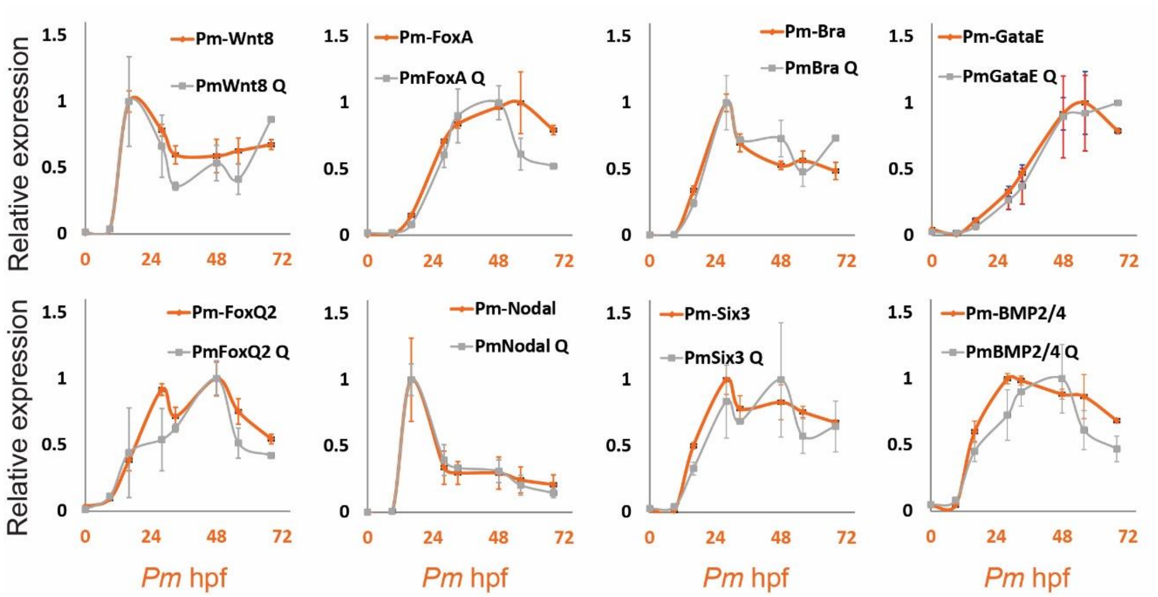

Figure S5 QPCR verification of RNA-seq results shows high agreement between the two measurements. Orange curves in each graph show RNA-seq results and grey lines show QPCR measurements at the same time points taken from [2]. Error bars correspond to standard deviation of three biological replicates in both experiments. Gene's name is indicated in each panel.

1. Barsi JC, Tu Q, Calestani C, Davidson EH. Genome-wide assessment of differential effector gene use in embryogenesis. Development. 2015;142(22):3892-901. doi: 10.1242/dev.127746. PubMed PMID: 26417044; PubMed Central PMCID: PMCPMC4712884.
2. Gildor T, Hinman V, Ben-Tabou-De-Leon S. Regulatory heterochronies and loose temporal scaling between sea star and sea urchin regulatory circuits. Int J Dev Biol. 2017;61(3-4-5):347-56. doi: 10.1387/ijdb.160331sb. PubMed PMID: 28621432.
